# Pain Assessment During Less Invasive Surfactant Administration Using Skin Conductance: A Prospective Cohort Study

**DOI:** 10.1038/s41390-025-04703-9

**Published:** 2025-12-22

**Authors:** Coralie Dirler, Vinzenz Boos, Dirk Bassler, Stephan Benke-Bruderer, Ulrike Held, Christoph M. Rüegger, Tobias Muehlbacher

**Affiliations:** 1 Newborn Research, Department of Neonatology, University Hospital Zurich, University of Zurich, Zurich, Switzerland; 2 Clinical Trials Center, University Hospital Zurich, Zurich, Switzerland; 3 Epidemiology, Biostatistics and Prevention Institute, University of Zurich, Zurich, Switzerland

## Abstract

**Background:**

Less Invasive Surfactant Administration (LISA) is an established treatment for surfactant deficiency. However, analgosedation for LISA remains controversial. The aim of this study was to assess pain using skin conductance (SC) measurements during LISA.

**Methods:**

This prospective cohort study included preterm infants born at ≥27 weeks. Pain was assessed using SC and blinded Neonatal Pain, Agitation and Sedation Scale (N-PASS) scoring. Primary outcome was change to baseline in SC peaks per second (PPS) at pre-specified events. Secondary outcomes included N-PASS scores and changes in cardiorespiratory parameters.

**Results:**

Twenty infants were included in the analysis. SC-PPS were increased during nasopharyngeal tube (NPT) insertion, but no more significant after adjusting for chronological age. SC-PPS were not increased at any other pre-specified event during LISA. N-PASS scores indicated elevated pain levels during NPT insertion (Δ_logit_ = 3.28, 95%-CI: 1.37 to 5.20) and laryngoscopy (Δ_logit_ = 3.60, 95%-CI 1.77 to 5.43). SC and N-PASS scores showed a weak correlation (Kendall’s τ_B_ = 0.17; 95%-CI: 0.02 to 0.31).

**Conclusion:**

LISA was not associated with elevated SC-PPS, despite increased N-PASS scores during specific procedural steps. This may reflect technical limitations of SC monitoring immediately after birth in preterm infants.

**Clinical Trial Registration:**

ClinicalTrials.gov (NCT06150586)

**Impact:**

- Skin conductance (SC) measurement has been proposed as an objective method for pain assessment in preterm infants. In our study, SC did not indicate a pain response in infants undergoing Less Invasive Surfactant Administration (LISA).
- Our findings suggest that SC alone may not be a reliable indicator of procedural pain in preterm infants shortly after birth.
- There remains a critical need for objective methods to assess pain in preterm infants. Further research is required to validate SC for neonatal pain assessment.

## Background

Methods of surfactant administration that avoid invasive mechanical ventilation are gaining popularity.^1–3^ Among these, Less Invasive Surfactant Administration (LISA) is a recommended approach for surfactant replacement therapy in preterm infants.^3–6^ Compared to surfactant administration via endotracheal tube, infants treated with LISA have a decreased risk of requiring mechanical ventilation, developing bronchopulmonary dysplasia (BPD) and for the composite outcome death or BPD.^1,5,7,8^

Although considered less invasive, LISA still requires laryngoscopy for the insertion of a catheter into the trachea, causing discomfort in some infants.^9,10^ While non-pharmacologic measures such as oral sucrose, swaddling and facilitated tucking are recommended,^11^ there is no consensus on the necessity of analgesic and/or sedative medication for LISA.^12,13^ A recent European survey revealed large differences in use and indication for such medication during the procedure.^2^

Pain assessment in preterm infants typically relies on validated multifactorial scoring systems such as the Neonatal Pain, Agitation and Sedation Scale (N-PASS),^14–17^ which represents the standard assessment tool at the University Hospital Zurich, where our study was conducted. Although the N-PASS is a reliable and recommended tool for this purpose, it remains susceptible to evaluator bias.^15^ Skin Conductance (SC) may be a more objective method for pain assessment. SC fluctuates with sympathetic activity, as eccrine sweat glands on the palms and soles are innervated by postganglionic sympathetic neurons. Sympathetic activation increases sweat secretion, reduces skin impedance, and subsequently, generates a SC peak, typically appearing 1-2 seconds after the corresponding stimulus. For pain assessment, changes in SC are quantified in peaks per second (PPS).^18^ While SC has been successfully used in several studies to assess procedural pain in preterm infants, their utility has not been explored in infants undergoing LISA, especially immediately after birth in the delivery room.^19–22^

The aim of this study was to assess pain in preterm infants during LISA using SC. Secondary aims included assessing pain with N-PASS scores and comparing these to SC-PPS, analyzing changes in cardiorespiratory parameters and investigating the occurrence of side effects during LISA and their effect on SC-PPS. We hypothesized that SC-PPS would increase during laryngoscopy, correlating with increased N-PASS values.

## Methods

This prospective cohort study was conducted at the Department of Neonatology of the University Hospital Zurich, between 12/2023 and 06/2024. All preterm infants meeting the local protocol criteria for LISA were eligible for inclusion: gestational age of ≥27 + 0 weeks, requiring non-invasive respiratory support with a positive end-expiratory pressure (PEEP) of ≥ 6 cmH_2_O and FiO_2_ ≥ 0.3 to maintain peripheral oxygen saturation (SpO_2_) of ≥ 90% for at least 15 minutes.

SC was continuously recorded using the MedStorm Pain-Sensor (MedStorm, Oslo, Norway). Three self-adhesive gel electrodes were placed on one foot of the infant for the duration of the study.^18^ The recorded data were automatically analyzed using an internal vendor software on a separate tablet computer. The algorithm converted the data into an ordinal scale from 0 (0 PPS) to 10 ( ≥ 0.40 PPS) at 1 Hz using a moving average over 15-second intervals. That scale correlates well with reported pain measured by numeric rating scale in adults.^23^ Heart rate (HR) and SpO_2_ were continuously measured by a Masimo pulse-oxymeter (Masimo Radical 7, Masimo Cooperation, Irvine, CA). Fraction of inspired oxygen (FiO_2_) was measured using an oxygen analyzer (AX300, Teledyne Analytical Instruments, California). These physiological parameters were recorded at 2 Hz intervals using the NewLifeBox™ system (Advanced Life Diagnostics UG, Weener, Germany). Each step of the LISA procedure was time-stamped on the tablet computer and a case report form. In addition, the entire LISA procedure was video recorded for blinded N-PASS assessment by two investigators (VB, CR), who had no access to the SC-PPS values.

Prior to the procedure, all patients received non-pharmacologic measures for pain relief (oral sucrose, swaddling and tucking). Caffeine citrate (20 mg/kg) was administered either immediately after birth or just prior to the LISA procedure. All infants received respiratory support via continuous positive airway pressure or nasal intermittent positive pressure ventilation by using the EVE NEO ventilator (Fritz Stephan GmbH, Gackenbach, Germany) with binasal prongs (Medijet, medin Medical Innovations GmbH, Olching, Germany) or a nasopharyngeal tube (NPT) (Portex, ICU Medical, San Clemente, CA or Vygon, Ecouen, France).

After the baseline recording prior to the LISA procedure, an NPT was inserted in all infants who had initially been stabilized with binasal prongs, as the Medijet interface extends four centimeters above the nose, hampering direct visualization of the larynx. LISA was performed by the attending physician on duty. The procedure involved laryngoscopy and insertion of the LISA catheter (Surfcath, Vygon, Ecouen, France), followed by the administration of 120-200 mg/kg of Surfactant (Curosurf, Chiesi Pharmaceuticals, Parma, Italy) over 30 to 90 seconds, and subsequent removal of the catheter. After a five-minute post-procedure stabilization period, infants were positioned prone and SC- and videorecording continued for sixty minutes ( ± 10 minutes) after LISA. The use of analgesic/sedative medication during the LISA procedure was permitted at the discretion of the attending physician.

The primary outcome was the change in median SC-PPS during predefined events compared to a 2-minute baseline period: insertion of the NPT, laryngoscopy, insertion of the LISA catheter, surfactant administration, removal of the LISA catheter (20-second periods), and 5 and 60 minutes following the LISA procedure (2-minute periods each).

Secondary outcomes included baseline-adjusted N-PASS pain scores (during laryngoscopy, surfactant administration, 5 and 60 minutes after LISA) and changes in cardiorespiratory parameters (HR, SpO₂, SpO₂/FiO₂ ratio). Furthermore, we collected information on adverse events associated with the LISA procedure (apnea lasting >10 seconds, desaturation <80% for >10 seconds, bradycardia <100/min for >10 seconds, surfactant reflux, and escalation of respiratory support) and their impact on SC-PPS. LISA failure (defined as the need for repeated LISA or intubation within one hour), occurrence of air leak syndrome or intraventricular hemorrhage (IVH) within 72 hours were recorded.

A post-hoc analysis included an additional blinded N-PASS assessment during NPT insertion, as this procedure elicited the most prominent behavioral signs of distress or pain in the infants.

The study including a deferred consent procedure was approved by the Local Ethics Committee (Kantonale Ethikkommission Zürich, BASEC-Nr. 2023-01602) and prospectively registered on ClinicalTrials.gov (NCT06150586). To safeguard the patient’s interests, initial consent for the study procedures was provided by an independent physician. Written informed consent was then obtained from all parents within 24 hours after the LISA procedure. In case of declined participation, all collected study data were destroyed.

The Palatin Foundation provided funding for MedStorm Pain-Sensor, salary (CD) and statistical analysis (SB, UH). The funders had no role in data collection, analysis, interpretation or manuscript writing.

### Statistical analysis

For descriptive analysis, continuous data were summarized using median and interquartile range, while categorical data were presented as counts and percentages of the total. Correlation between SC-PPS and N-PASS scores was assessed using Kendall rank correlation τ_B_, and between SC-PPS and physiological parameters using Spearman’s rank correlation. Mixed-effects regression models were employed to analyze repeated measurements of the outcomes over time with a random intercept for each participant and median measurement values over the event durations as response variable (aggregated data). The use of linear mixed models (LMM) had been planned for all measurement variables, but the high proportion of zero values for SC measurements and N-PASS values required a change to cumulative link mixed models (CLMM) for ordinal regression for these variables. Time was treated as a factor variable, corresponding to the pre-defined events in the procedure. The effect of each event was determined by taking the difference of the estimated event marginal mean to that of the baseline (on the linear predictor scale, see Supplementary) and reported with 95% confidence intervals. For the LMMs, contrasts were tested using *t*-tests and Kenward’s method^24^ to estimate the degrees of freedom for the tests and CIs. For the CLMMs, contrasts were tested using *z*-tests and the asymptotic normal approximation for the tests and CIs. Within one set of comparisons, *p*-values were adjusted for the multiple comparisons to baseline using an approximation of Dunette’s method.

All statistical analyses were conducted using R Version 4.4.1 (R Core Team (2024))^25^ and the following R packages: lme4 for the LLMs, ordinal for the CLMMs, emmeans for the estimated marginal means and DescTools for the correlations. Data was processed using the tidyverse packages and figures created using ggplot2 and flowchart for the participant flowchart.

Gestational age, chronological age at LISA and sedative medication were predefined as potential confounding variables and sensitivity analyses were carried out for these variables by including them and their interaction with time in the models. Due to the limited amount of datapoints, separate models were fitted for each variable. Statistical significance was defined as *p*-values < 0.05. Due to lack of information on SC values in this specific cohort, no formal sample size calculation could be performed. A prospectively collected convenience sample of 20 infants was included to ensure feasibility and provide sufficient clinically relevant data.

The reporting of this observational study followed the consensus-based Strengthening the Reporting of Observational Studies in Epidemiology (STROBE) guidelines.^26^

## Results

**Fig. 1:**
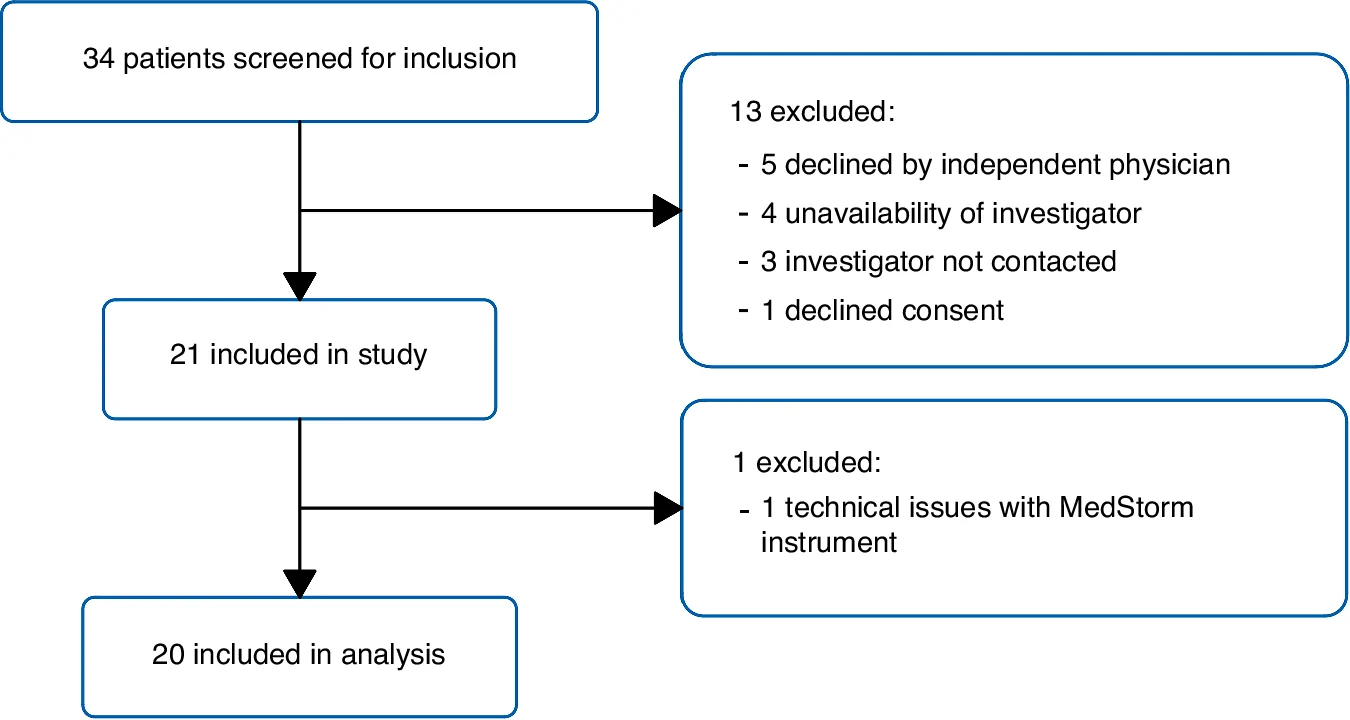
Flowchart of screened and included infants.

A total of twenty infants were included in the final analysis (Fig. 1). Their demographic and clinical characteristics are summarized in Table 1.

**Table 1:** Baseline patient characteristics.

Demographic data	Median [IQR], *n* (%)
Gestational age at birth (weeks)	30.00 [28.1, 30.8]
Birth weight (g)	1300 [1058, 1723]
Female sex	8 (40.0%)
**Mode of delivery**	
Cesarean section	17 (85.0%)
Vaginal delivery	3 (15.0%)
**Antenatal corticosteroids**	
None	1 (5.0%)
Incomplete ( < 48 h)	9 (45.0%)
Complete	10 (50.0%)
**APGAR**	
5’	6.5 [5.0, 8.0]
10’	9.0 [9.0, 9.0]
**Clinical data**	
Respiratory support before LISA	
CPAP	16 (80.0%)
NIPPV	4 (20.0%)
PEEP level	8.0 [8.0, 8.0]
Hours of life at LISA procedure (chronological age)	1.65 [0.57, 4.06]
Duration of the LISA procedure (minutes)^a^	8.1 [IQR 4.4, 10.8]
Partial surfactant reflux	5 (25.0%)
Escalation of respiratory support	8 (40.0%)
Use of sedative medication	5 (25%)

Depicted as median and interquartile range or *n* (%).

*CPAP* continuous positive airway pressure, *NIPPV* nasal intermittent positive pressure ventilation, *PIP* Peak Inspiratory Pressure, *PEEP* Positive End-Expiratory Pressure.

^a^time from baseline-measurement until removal of LISA catheter.

**Fig. 2:**
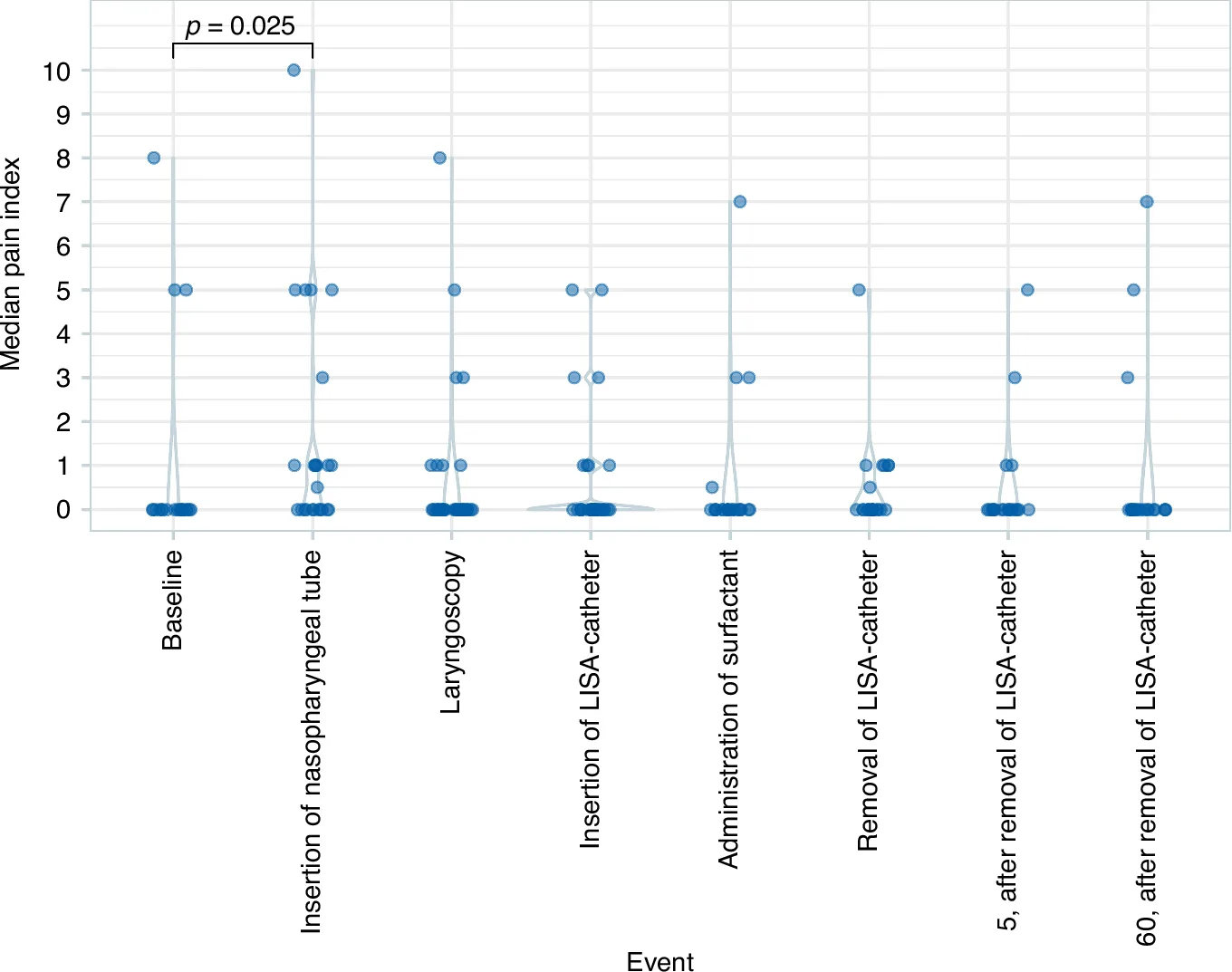
Distribution of SC pain index across all steps of the LISA procedure. The SC pain index is calculated by an internal vendor software algorithm resulting in an ordinal scale from 0 = 0 peaks-per-second (PPS) to 10 = ≥ 0.4 PPS. The grey lines are violin plots of the data. Brackets and *p*-values indicate timepoints with significant differences to baseline (Table 2).

Figure 2 illustrates the distribution of SC-PPS across all events of the LISA procedure. Apart from a significant increase in SC-PPS during NPT insertion (Δ_logit_= 2.38, 95%-CI: 0.20 to 4.55), none of the other procedural steps were associated with elevated SC-PPS compared to baseline (Table 2). Chronological age emerged as a potential confounder for SC-PPS with a significant interaction in the ordinal regression model with higher chronological age being associated with lower SC-PPS values mainly during baseline and after LISA. No interactions with other confounders, such as gestational age or analgosedation, were identified and no evidence for a difference in SC-PPS or N-PASS values compared to baseline was observed between non-sedated and sedated infants (Supplementary Figs. S1 and S2).

**Table 2:** Estimated differences from baseline (Δ_logit_, linear predictor scale) in SC measurements and N-PASS across all steps of the LISA procedure, based on ordinal regression models.

Contrast	Skin Conductance			N-PASS		
	Estimated logit difference	95% CI	*p*-value	Estimated logit difference	95% CI	*p*-value
insertion of NPT—Baseline	2.375	0.198 to 4.551	0.025	3.2839	1.3658 to 5.2021	<0.0001
laryngoscopy—Baseline	0.693	−1.503 to 2.889	0.88	3.6020^a^	1.7749 to 5.4291^a^	<0.0001
insertion of LISA catheter—Baseline	0.660	−1.555 to 2.875	0.90			
administration of surfactant—Baseline	-0.001	−2.477 to 2.475	1.00	1.8955	−0.0360 to 3.8271	0.057
removal of LISA catheter—Baseline	0.584	−1.715 to 2.883	0.94			
5’ after removal of LISA catheter—Baseline	0.025	−2.454 to 2.505	1.00	0.4658	−1.4700 to 2.4017	0.92
60’ after removal of LISA catheter—Baseline	-0.131	−2.695 to 2.432	1.00	−1.8470	−4.4210 to 0.7270	0.25

Confidence intervals are asymptotic; *p*-values are adjusted for multiple comparisons. ^a^values of the ordinal regression model after the post-hoc inclusion of N-PASS scores during insertion of the NPT.

Confidence intervals are asymptotic; *p*-values are adjusted for multiple comparisons.

^a^values of the ordinal regression model after the post-hoc inclusion of N-PASS scores during insertion of the NPT.

**Fig. 3:**
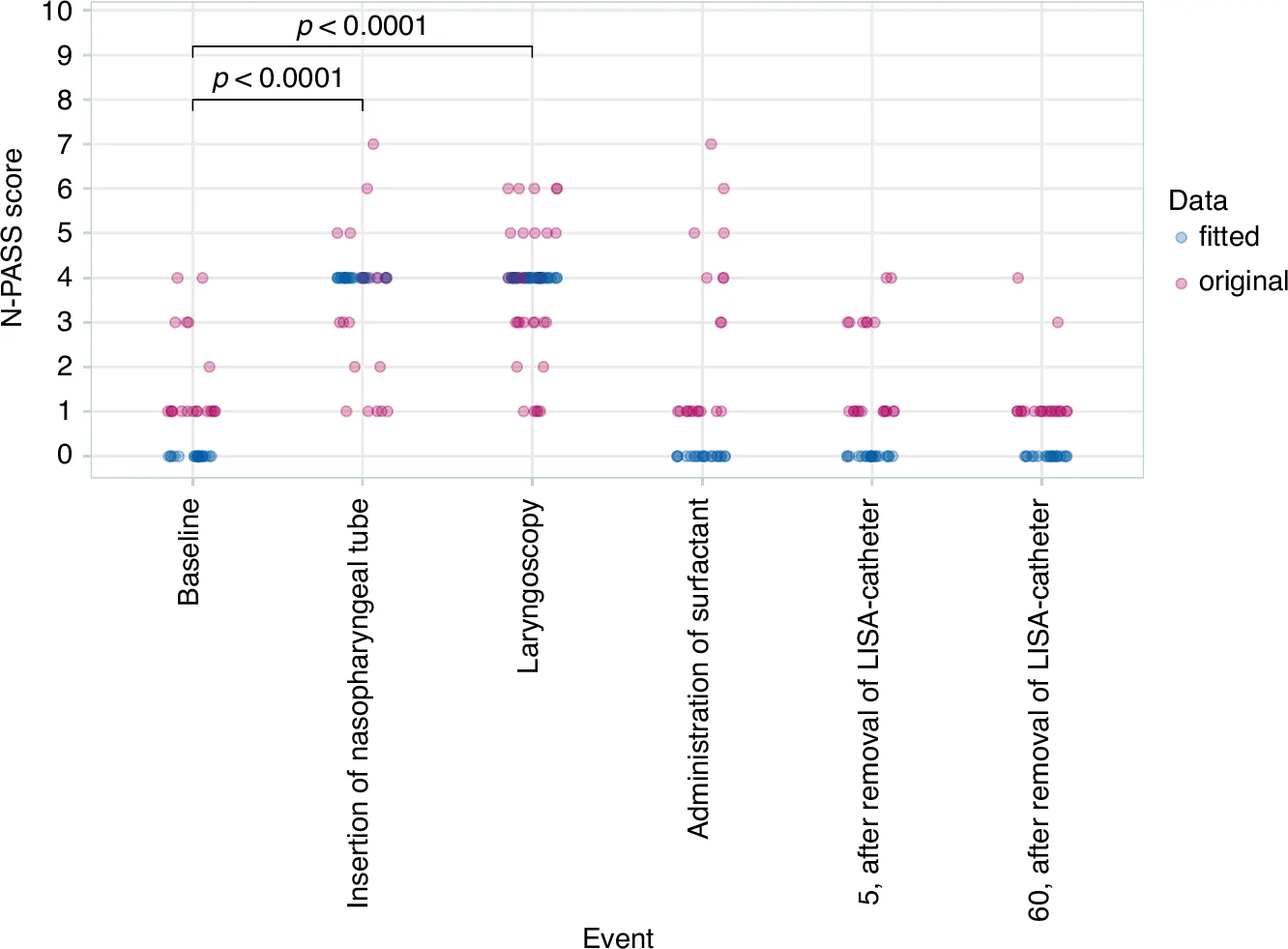
Comparison of original data with fitted values of N-PASS scores from the ordinal regression model during defined LISA steps. Brackets and *p*-values indicate timepoints with significant differences to baseline (Table 2).

The ordinal regression model showed no increase in estimated differences for N-PASS scores for all procedural steps except laryngoscopy (Δ_logit_ = 3.36, 95%-CI 1.59 to 5.12, Table 2, Fig. 3).

During LISA, changes in cardiorespiratory parameters could be observed. Compared to baseline, HR decreased during surfactant administration and removal of the LISA catheter (Supplementary Fig. S3). SpO_2_ was lower during insertion of the NPT (Supplementary Fig. S4). During insertion of the LISA catheter, surfactant administration and removal of the LISA catheter both, SpO_2_ and SpO_2_/FiO_2_ ratio were lower (Supplementary Fig. S4 and Supplementary Fig. S5). The SpO_2_/FiO_2_ ratio showed an increase at 5 and 60 minutes after catheter removal (Supplementary Fig. S5).

LISA failure occurred in three infants (15%), all of whom required intubation within 72 hours. Apnea was observed in three infants (15%, five events), desaturation in 11 infants (55%, 21 events), and bradycardia in five infants (25%, six events). None of these had a measurable impact on SC-PPS. Two infants (10%) developed a pneumothorax, and three (15%) were diagnosed with IVH grade I at the first ultrasound scan after 72 hours of life.

The primary analysis revealed no significant correlation between SC-PPS and N-PASS scores (Kendall’s τ_B_ = 0.10, 95%-CI: -0.07 to 0.27). Additionally, no correlations were identified between SC-PPS and any cardiorespiratory parameter.

### Post-hoc analysis

The ordinal regression model of the N-PASS showed a significant increase from baseline during NPT insertion (Δ_logit_ = 3.28, 95%-CI: 1.37 to 5.20, Table 2, Fig. 3). Inclusion of the additional values resulted in a slightly higher increment from baseline for laryngoscopy in the ordinal regression model (Δ_logit_ = 3.60, 95%-CI: 1.77 to 5.43, Table 2, Fig. 3) and in an overall positive correlation between SC-PPS and N-PASS scores (Kendall’s τ_B_ = 0.17, 95%-CI: 0.02 to 0.31).

Graphical analysis showed no evidence for a difference in SC-PPS when comparing infants receiving NPT insertion during LISA to those in whom NPT was placed prior to baseline (Supplementary Fig. S6). A smaller change from baseline in N-PASS values is visible in infants with NPT insertion prior to baseline (Supplementary Fig. S7). Infants with NPT prior to baseline had higher N-PASS values at baseline (Supplementary Fig. S8). No further statistical analysis was performed due to the low number of events.

## Discussion

The primary aim of this study was to assess pain during the LISA procedure using SC measurements. While a significant increment in pain-related SC was initially observed during NPT insertion, no other procedural steps—including laryngoscopy—demonstrated significant SC changes. Following adjustment for chronological age as a potential confounding variable, SC during NPT insertion was no longer significantly different from baseline values. This may be a statistical artifact, possibly arising from the addition of another parameter to an already complex model with limited data points, particularly since N-PASS scores simultaneously indicate elevated pain during this specific procedure.

The absence of a robust SC response observed in our study may be attributed to several factors. First, there was marked inter-individual variability in SC responses, which may reflect technical challenges inherent to SC measurement in preterm infants. Similar variability in PPS values has been reported in previous studies during neonatal intubation.^27^ Notably, those studies observed elevated baseline PPS values, which were not present in our cohort. Second, temporal alignment between SC responses and procedural events was inconsistent. In several instances, the standardized time windows captured only part of the SC response or increases in PPS occurred outside the predefined event windows. This would be consistent with previous findings of spontaneous PPS elevations occurring in the absence of identifiable external stimuli.^27^

The reliability of SC in detecting pain responses in preterm infants has been demonstrated in previous studies, with measurable responses observed as early as 22 weeks of gestational age.^19,28^ However, these studies measured SC between a median postnatal age of 7 days and 5 weeks.^29^ In contrast, the median postnatal age in our study was 1.65 hours, representing the earliest time point after birth at which SC has been evaluated. The unique physiological conditions present shortly after birth may impair SC measurements and may have resulted in a high proportion of SC measurements without detectable SC-PPS. Second, early SC measurements performed immediately after birth may have been influenced by elevated baseline skin conductivity, attributable to increased skin water content and skin immaturity.^30,31^ The early postnatal timing of measurements in our study may have compromised the sensitivity of SC to detect impedance changes caused by sympathetic activation and sweat gland stimulation, particularly in response to mild noxious stimuli. However, due to the limited sample size for the complex model, our study might lack the necessary power to show an effect.

We also observed substantial discrepancies between pain assessment based on SC and N-PASS scores. Blinded assessment of pain using the N-PASS revealed a significant increase in scores during laryngoscopy, consistent with previous studies reporting elevated distress during the LISA procedure.^32,33^ However, the absence of a corresponding increase in SC suggests that laryngoscopy, while subjectively perceived as painful, may not be a sufficiently intense noxious stimulus to provoke detectable autonomic responses as measured by SC. Furthermore, the observed discrepancies may reflect limitations in the validity of behavioral pain assessments, as the ability to discriminate between noxious and non-noxious stimuli depends on brain maturation and typically emerges only in preterm infants beyond 33 weeks of gestation.^34,35^ Therefore, we cannot rule out the possibility of evaluator bias when using the N-PASS to assess discomfort during non-noxious or only mildly noxious stimuli, as this may occur without a corresponding SC response, resulting in the observed weak correlation between SC and N-PASS. Other studies have reported a stronger correlation between SC and behavioral pain assessments in preterm but chronologically older infants.^20,36^

While N-PASS scores indicated procedural distress, HR remained stable throughout the procedure. As noxious-evoked increases in HR are known to be influenced by neurological maturation,^35^ the absence of a HR response in our more premature cohort may reflect an immature autonomic response to pain. A decrease in SpO_2_ was observed during both NPT and LISA catheter insertion. We attribute this to interrupted PEEP delivery during prong removal for NPT placement, and later to partial glottic obstruction by the LISA catheter.^9^ Laryngoscopy itself had no measurable effect on SpO_2_ or HR, despite the potential for vagal stimulation and/or significant PEEP loss.^9^ Between the onset of surfactant administration and removal of the LISA catheter, HR and SpO_2_ decreased significantly. This response is likely attributable to transient airway obstruction caused by the intratracheally instilled surfactant, as well as potential activation of the laryngeal chemoreflexes with vagal stimulation from the administration of surfactant.^37,38^ The SpO_2_/FiO_2_ ratio demonstrated a similar trajectory, declining during the procedure followed by a rapid rise above baseline thereafter, indicating a beneficial effect of successful surfactant administration on oxygenation. Notably, neither N-PASS scores nor SC values increased during this phase, suggesting the absence of significant noxious stimulation during surfactant delivery.

During the study, the strongest behavioral response was observed during insertion of the NPT. Consequently, blinded N-PASS scores for this procedural step were included in a post-hoc analysis. This analysis was not pre-specified in the original study protocol, because NPT insertion is not a routine component of the LISA procedure in many other NICUs.^6,39^ In 18 out of 20 infants, the NPT was inserted as part of the LISA procedure. Two infants had already undergone NPT insertion prior to baseline. There was no difference in SC-PPS compared to baseline between infants with prior NPT and those in whom NPT was inserted during LISA. However, the change in N-PASS values from baseline appeared smaller in infants with prior NPT, likely due to higher baseline N-PASS values reflecting ongoing discomfort from the prior painful stimulus. However, this finding should be interpreted with caution, as only two infants had NPT insertion prior to baseline, and this should be addressed in future investigations. Interestingly, during NPT insertion, a positive correlation was observed between N-PASS scores and SC-PPS values, suggesting a particularly strong pain response compared to all other steps of the procedure.

Our study has several limitations. First, the small sample size limits statistical power and generalizability. No conclusion can be drawn for more premature infants, as our LISA protocol does not include infants <27 weeks of gestation. Second, the variability of SC responses across participants, as well as spontaneously elevated PPS values observed outside of the LISA procedure may complicate the interpretation of the SC data. Third, practical challenges affected blinded N-PASS scoring. For example, facial expressions were frequently obscured by the clinician’s hands, and assessment of extremity tone was limited in swaddled infants. Fourth, due to a high frequency of zero PPS values, the originally planned linear mixed model had to be replaced with an ordinal regression approach which also has limitations with aggregation of zero values. Finally, the correlation of SC and N-PASS scores only reached statistical significance in a post hoc analysis following the inclusion of N-PASS data from NPT insertion.

## Conclusion

In this study, there was no evidence that SC responded to procedural actions associated with potential pain sensation during the LISA procedure. Increased N-PASS values indicate pain and/or discomfort during laryngoscopy.

The weak correlation between SC and behavioral pain scores indicates that SC alone may not have been a reliable indicator of procedural pain at this early developmental stage. Further research is needed to validate SC as a tool for neonatal pain assessment.

## Supplementary information

Supplemental material

Supplemental Table 1

## Data Availability

The datasets generated during and/or analysed during the current study are available from the corresponding author on reasonable request.
