# Supplementary material for: Pain Assessment During Less Invasive Surfactant Administration Using Skin Conductance: A Prospective Cohort Study
Source: Pediatr Res. 2025 Dec 22;100(2):913–9. doi: 10.1038/s41390-025-04703-9 (PMC13638450; doi:10.1038/s41390-025-04703-9)
Supplement: Supplementary file 1 [file 41390_2025_4703_MOESM1_ESM.pdf]

Supplemental material

Supplemental Figure S1. Comparison of difference of SC-pain index to baseline in infants receiving no analgosedation before the respective event vs. those who were treated with analgosedative medication.

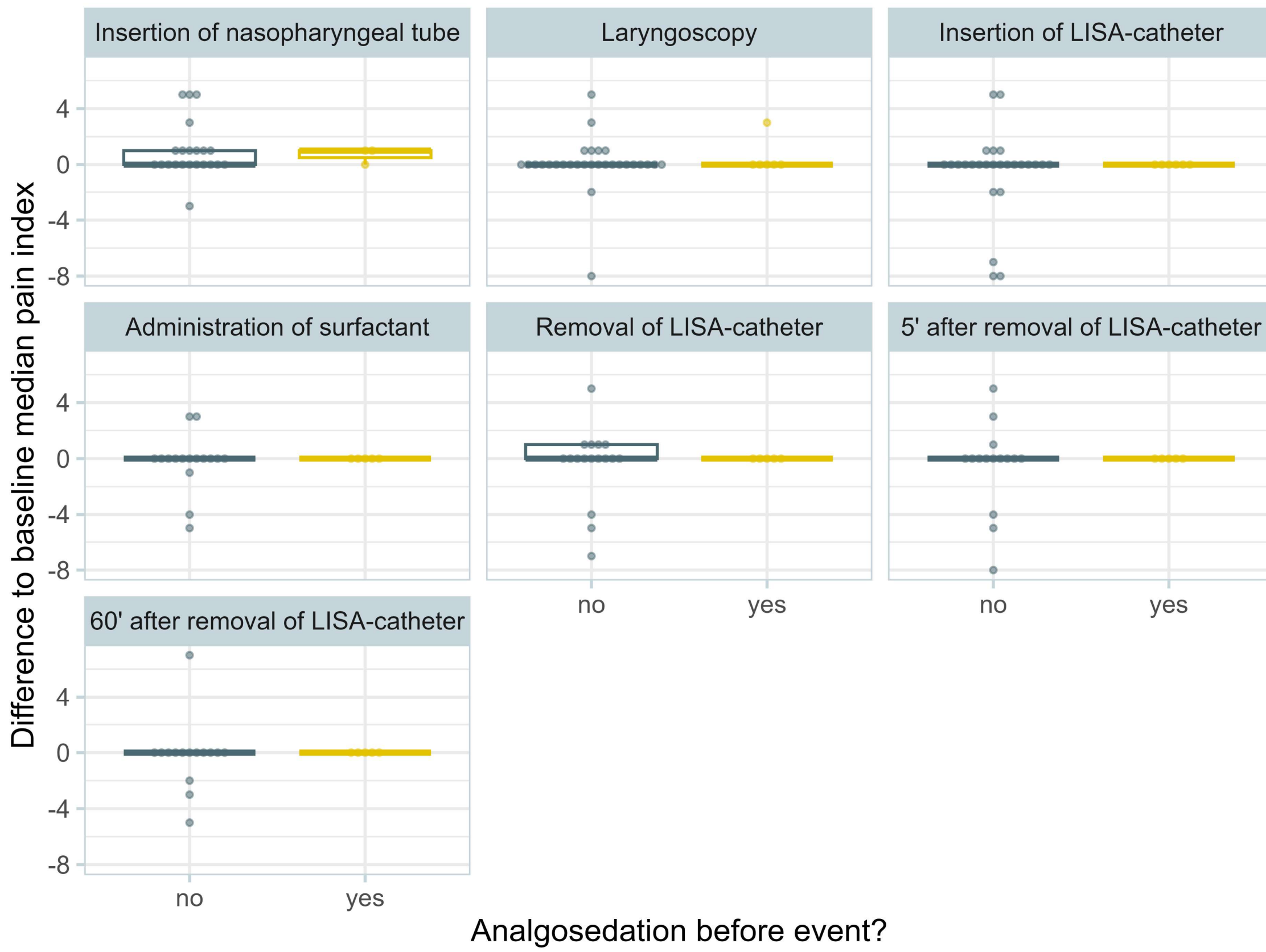

Supplemental Figure S2. Comparison of difference of N-PASS to baseline N-PASS in infants receiving no analgosedation before the respective event vs. those who were treated with analgosedative medication.

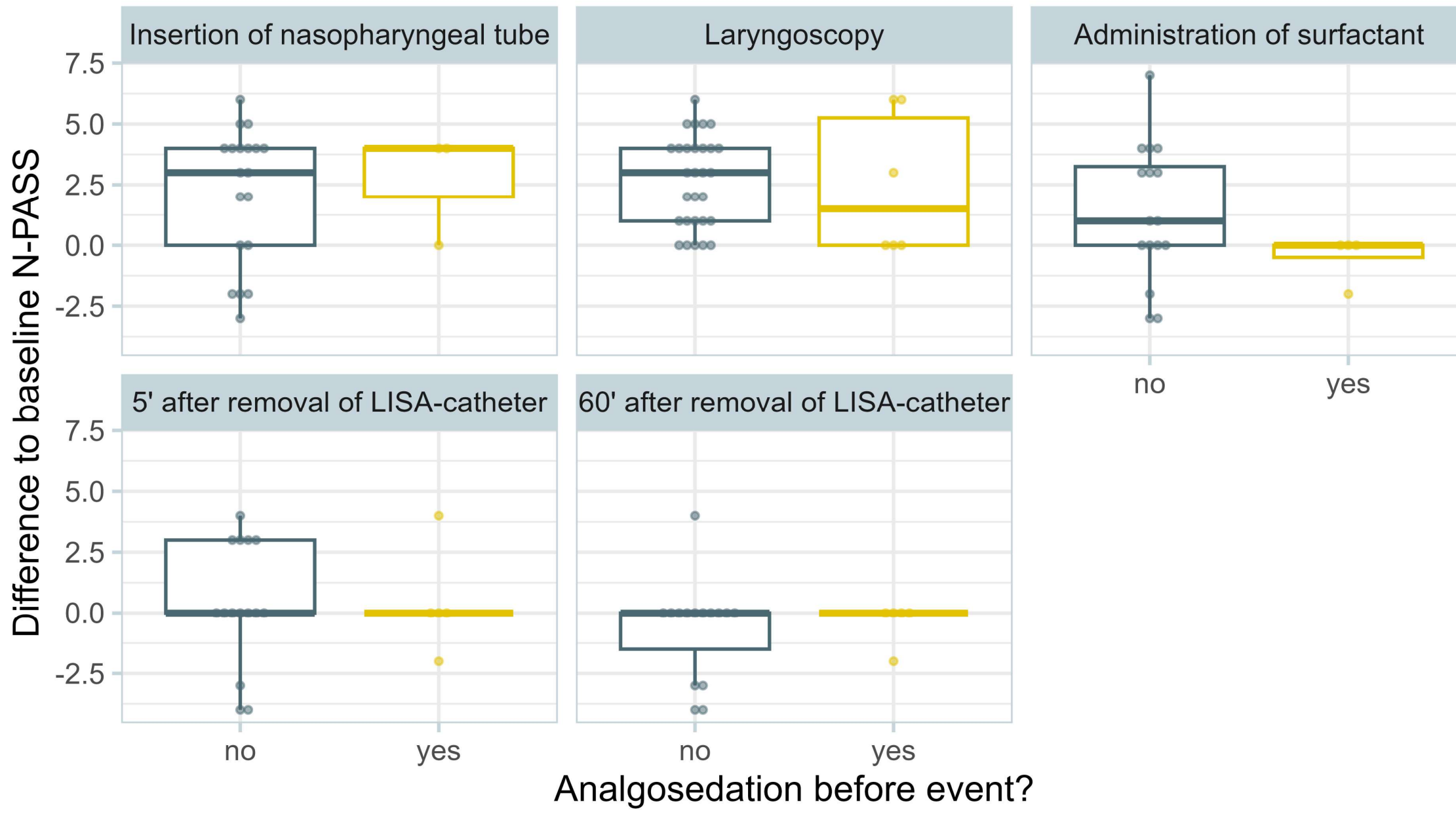

Supplemental Figure S3. Comparison of original heart rate data with linear mixed model predictions across LISA steps. Orange bars represent estimated marginal means (i.e., across random effects) with 95% confidence interval calculated using the Kenward-Roger method). Brackets and p-values indicate timepoints with significant differences to baseline.

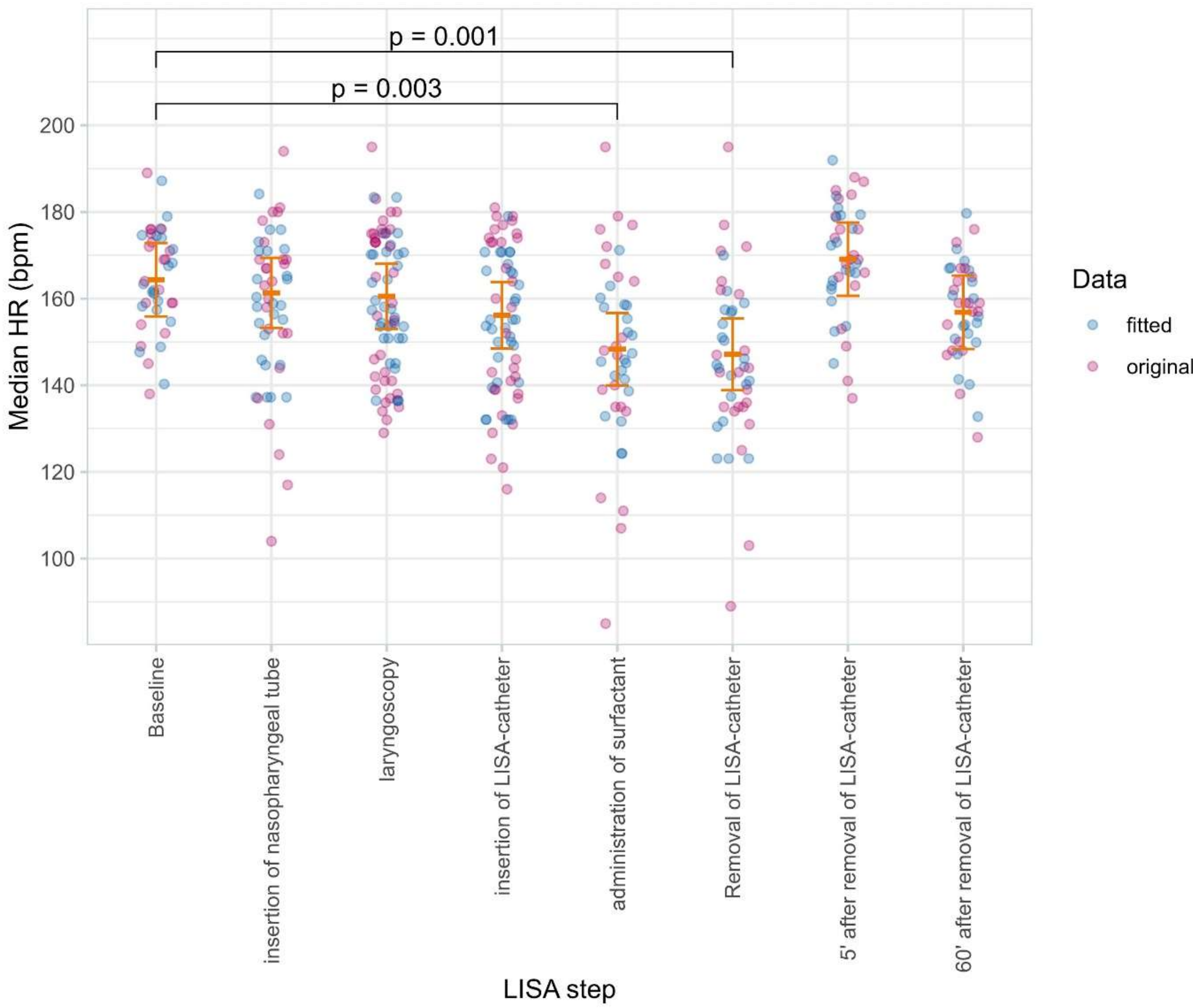

Supplemental Figure S4. Comparison of original SpO_2 data with linear mixed model predictions across LISA steps. Orange bars indicate estimated marginal means (i.e., across random effects) with 95% confidence intervals computed using the Kenward-Rogers method). Brackets and p-values indicate timepoints with significant differences to baseline.

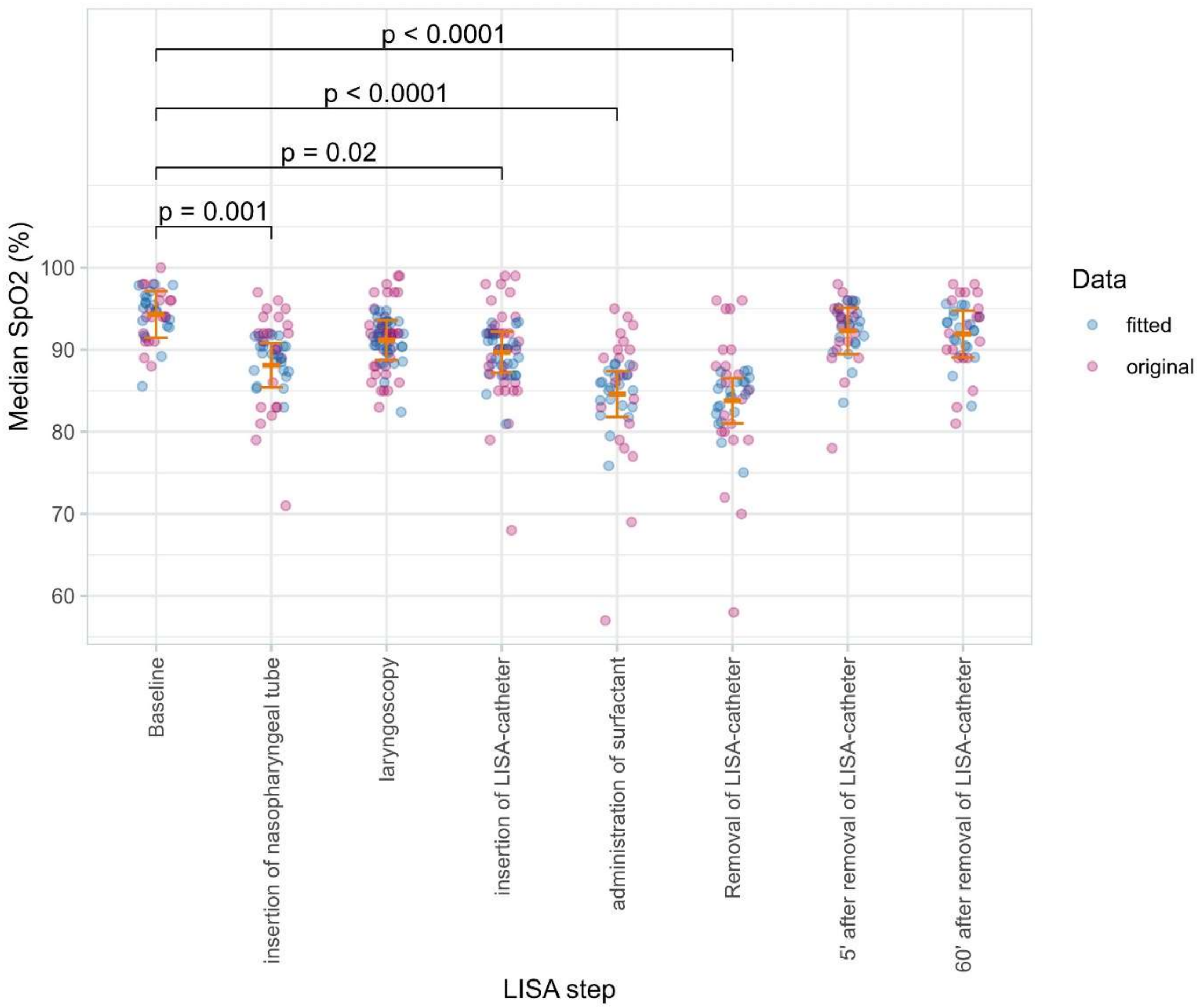

Supplemental Figure S5. Comparison of original SpO_2/FiO_2 data and linear mixed model predictions across LISA steps. Orange bars represent estimated marginal means (i.e., across random effects) 95% confidence intervals calculated using the Kenward-Rogers method). Brackets and p-values indicate timepoints with significant differences to baseline.

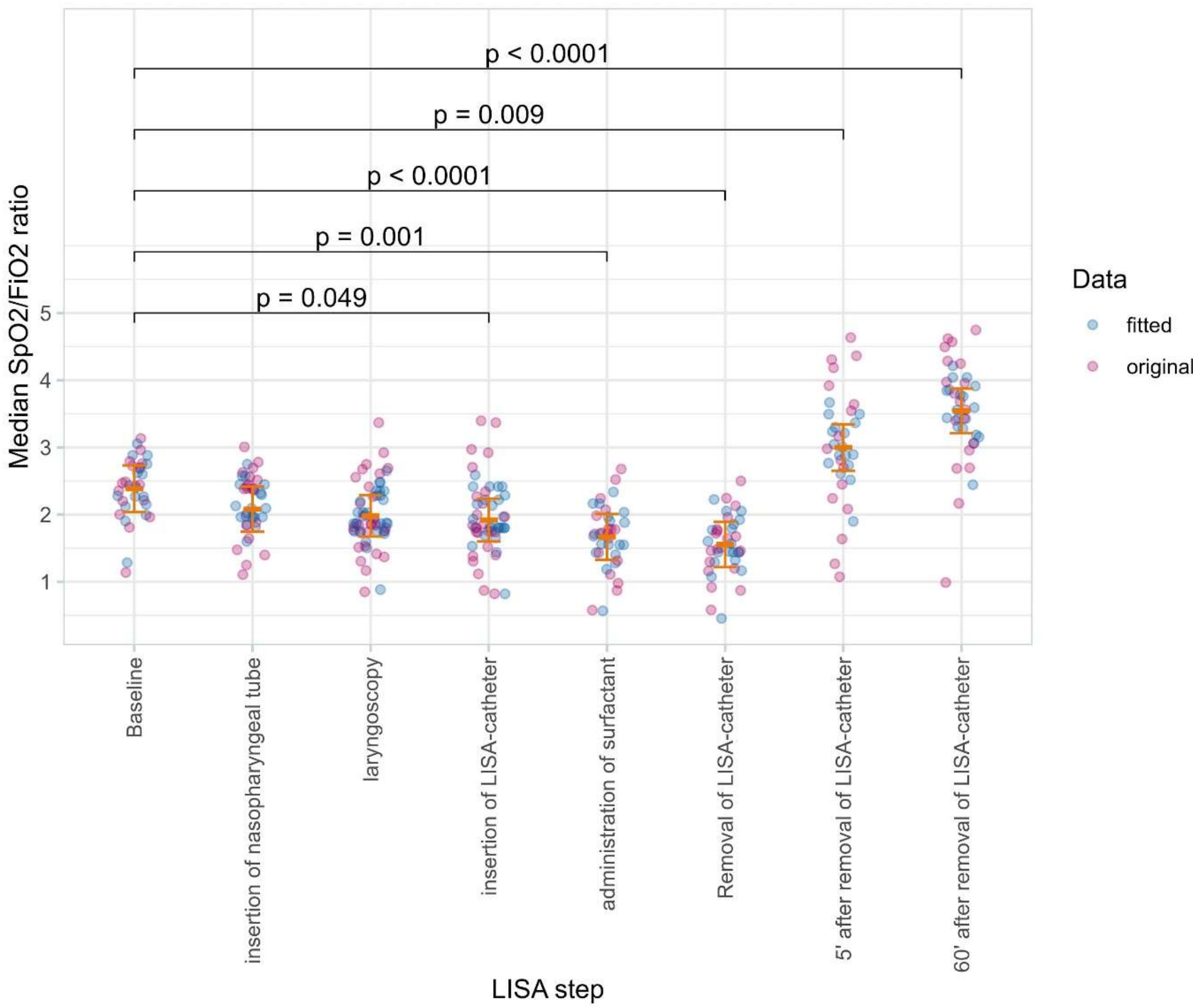

Supplemental Figure S6. Graphical analysis of SC pain index in infants who received NPT insertion as part of the LISA procedure (turquoise) compared to those, in whom NPT was placed before baseline (orange). Comment on left-top panel "insertion of nasopharyngeal tube": dislocation of the NPT during LISA required re-insertion of the NPT, therefore there are few existing datapoints even for that event.

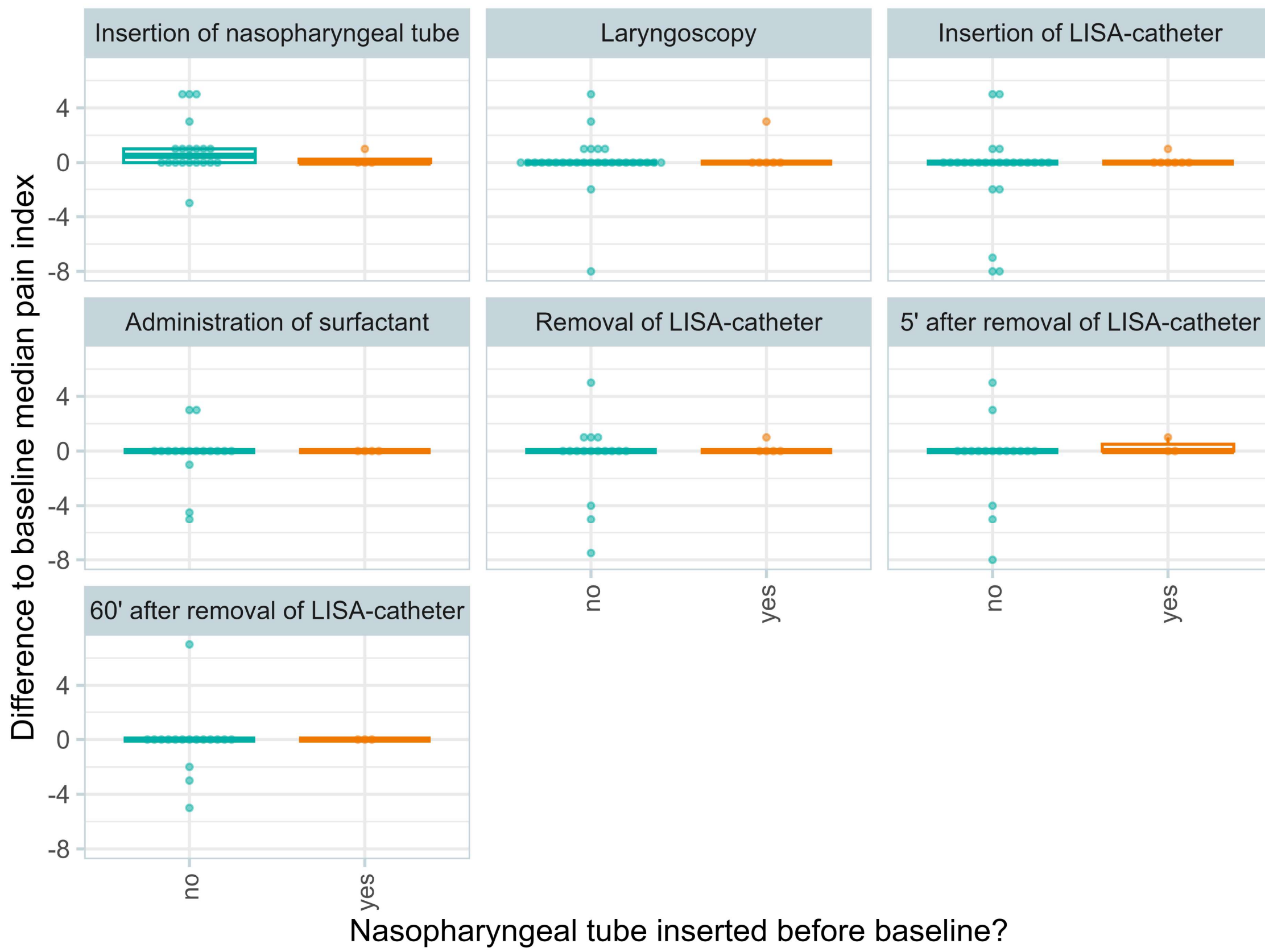

Supplemental Figure S7. Graphical analysis of N-PASS in infants who received NPT insertion as part of the LISA procedure (turquoise) compared to those, in whom NPT was placed before baseline (orange). Comment on left-top panel "insertion of nasopharyngeal tube": dislocation of the NPT during LISA required re-insertion of the NPT, therefore there are few existing datapoints even for that event.

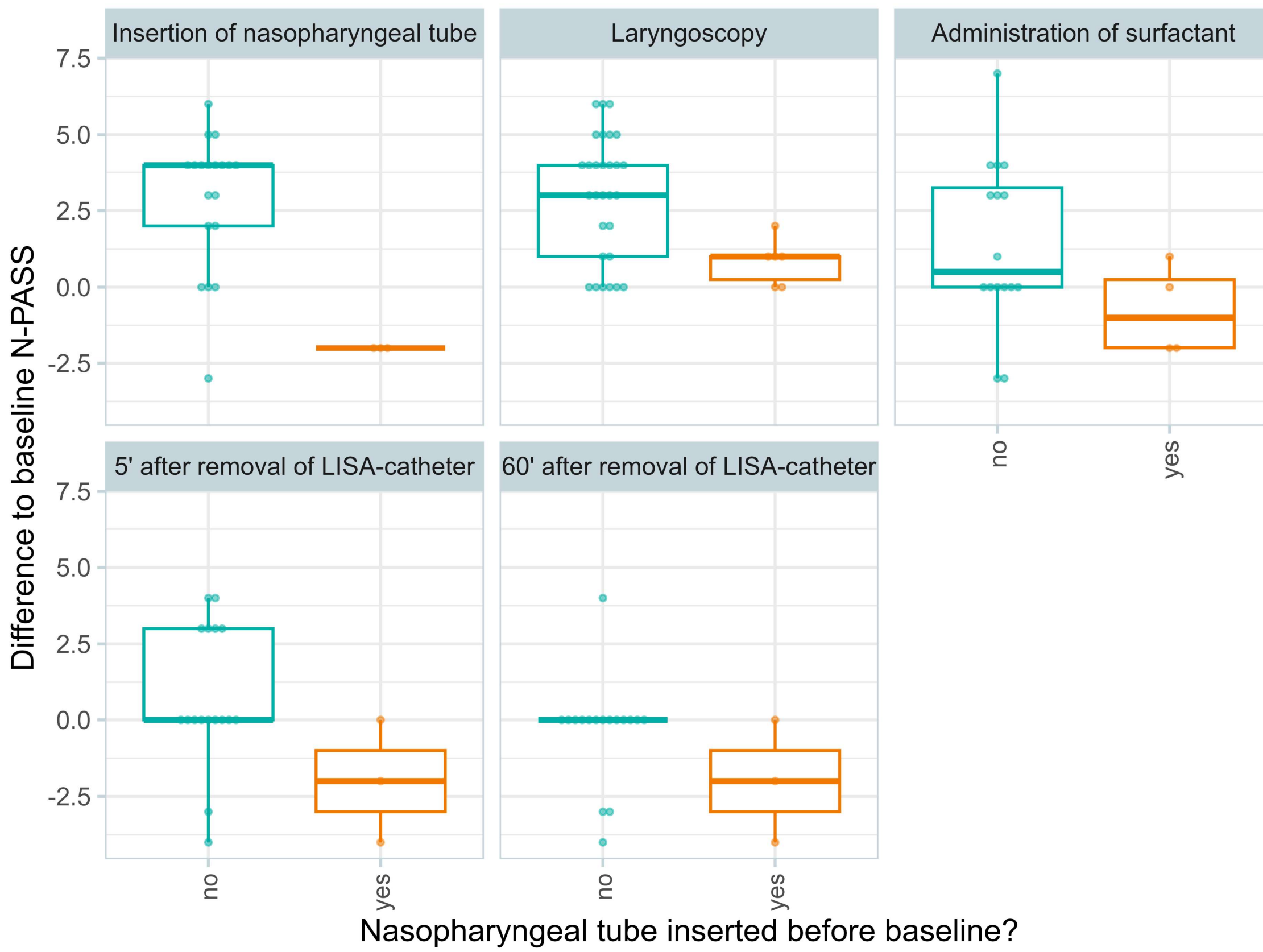

Supplemental Figure S8. Graphical analysis of N-PASS at baseline in infants who received NPT insertion as part of the LISA procedure (turquoise) compared to those, in whom NPT was placed before baseline (orange).

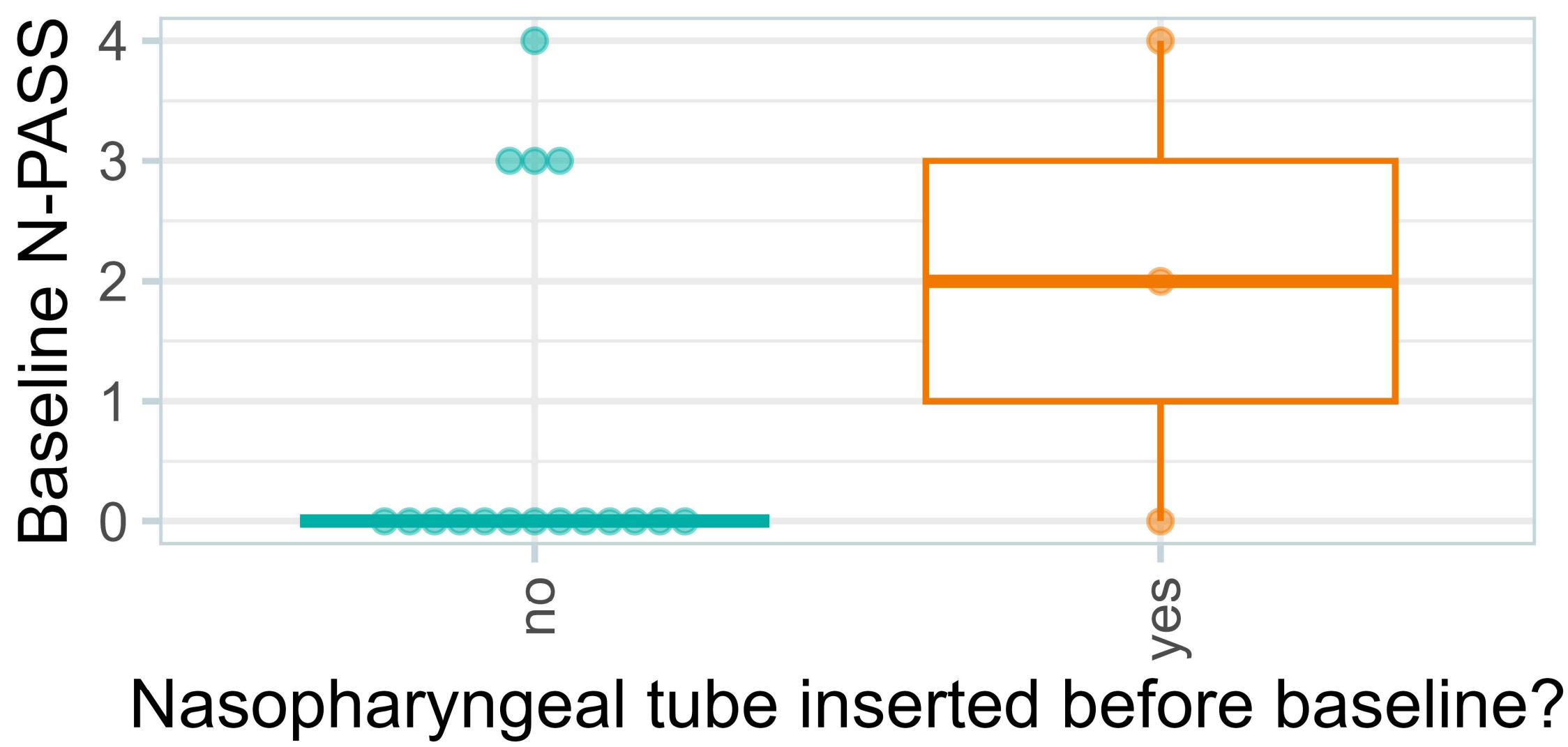

Supplement: additional information for statistical modeling and references for used R-packages:

With the unexpected high proportion of 0 values in both the SC and N-PASS data, linear mixed models did not produce sensible results anymore. We therefore decided to switch to ordinal regression using cumulative link mixed models (CLMM) with a logit link function. While the CLMM are better suited for the data at hand, they come at the price of higher model complexity (as the model must additionally fit thresholds for the categories of the response variable) and are less intuitively understandable.

In a linear model, the predictor scale corresponds directly to the scale of the response variable. For example, in a linear model with N-PASS as the response variable, the differences of the estimated marginal for the different time points can directly be interpreted as differences in N-PASS scores. In an ordinal regression model, such a straightforward interpretation is not possible. For the CLMM, there are no N-PASS values but ordered categories of N-PASS scores. The model then predicts the probability of a data point falling into a certain category.

It is therefore important to note that the differences in marginal means we report in this paper for the CLMMs are on the logit predictor scale of the models (Δ_{logit}) and cannot be interpreted directly in terms of differences in SC or N-PASS values. That the Δ_{logit} values for the N-PASS model are in a similar range as the N-PASS values is pure coincidence.

lme4	Bates D, Mächler M, Bolker B, Walker S. Fitting Linear Mixed-Effects Models Using lme4.
ordinal	RHB C. ordinal - Regression Models for Ordinal Data: R Foundation for Statistical Computing; 2022 [2022.11-16:[Available from: https://CRAN.R-project.org/package=ordinal.
emmeans	RV L. emmeans: Estimated Marginal Means, aka Least-Squares Means 2024 [1.10.1:[Available from: https://CRAN.R-project.org/package=emmeans.
DescTools	Signorell A ea. DescTools: Tools for Descriptive Statistics 2024 [0.99.52:[Available from: https://CRAN.R-project.org/package=DescTools.
tidyverse	al. We. Welcome to the Tidyverse. Journal of Open Source Software. 2020;4(43).
ggplot2	H W. ggplot2: Elegant Graphics for Data Analysis. New York: Springer-Verlag; 2016.
flowchart	Satorra P CJ, Pallarès N, Tebé C. flowchart: Tidy Flowchart Generator. 2024.
